# A rice mTERF protein V14 sustains photosynthesis establishment and temperature acclimation in early seedling leaves

**DOI:** 10.1186/s12870-021-03192-2

**Published:** 2021-09-06

**Authors:** Man Wang, Feng Zhou, Hong Mei Wang, De Xing Xue, Yao-Guang Liu, Qun Yu Zhang

**Affiliations:** 1 Present Address: State Key Laboratory for Conservation and Utilization of Subtropical Agro-bioresources, College of Life Sciences, South China Agricultural University, Guangzhou, 510642 China; 2 Guangdong Laboratory for Lingnan Modern Agriculture, Guangzhou, 510642 China; 3 SCAU Main Campus Teaching & Research Base, Guangzhou, China

**Keywords:** mTERFs, *rpoB*-*rpoC1*-*rpoC2* operon, Chloroplast development, Temperature response, Rice

## Abstract

**Background:**

Plant mitochondrial transcription termination factor (mTERF) family members play important roles in development and stress tolerance through regulation of organellar gene expression. However, their molecular functions have yet to be clearly defined.

**Results:**

Here an mTERF gene *V14* was identified by fine mapping using a conditional albino mutant *v14* that displayed albinism only in the first two true leaves, which was confirmed by transgenic complementation tests. Subcellular localization and real-time PCR analyses indicated that *V14* encodes a chloroplastic protein ubiquitously expressed in leaves while spiking in the second true leaf. Chloroplastic gene expression profiling in the pale leaves of *v14* through real-time PCR and Northern blotting analyses showed abnormal accumulation of the unprocessed transcripts covering the *rpoB*-*rpoC1* and/or *rpoC1*-*rpoC2* intercistronic regions accompanied by reduced abundance of the mature *rpoC1* and *rpoC2* transcripts, which encode two core subunits of the plastid-encoded plastid RNA polymerase (PEP). Subsequent immunoblotting analyses confirmed the reduced accumulation of RpoC1 and RpoC2. A light-inducible photosynthetic gene *psbD* was also found down-regulated at both the mRNA and protein levels. Interestingly, such stage-specific aberrant posttranscriptional regulation and *psbD* expression can be reversed by high temperatures (30 ~ 35 °C), although *V14* expression lacks thermo-sensitivity. Meanwhile, three *V14* homologous genes were found heat-inducible with similar temporal expression patterns, implicating their possible functional redundancy to *V14*.

**Conclusions:**

These data revealed a critical role of V14 in chloroplast development, which impacts, in a stage-specific and thermo-sensitive way, the appropriate processing of *rpoB*-*rpoC1*-*rpoC2* precursors and the expression of certain photosynthetic proteins. Our findings thus expand the knowledge of the molecular functions of rice mTERFs and suggest the contributions of plant mTERFs to photosynthesis establishment and temperature acclimation.

**Supplementary Information:**

The online version contains supplementary material available at 10.1186/s12870-021-03192-2.

## Background

The mitochondrial transcription termination factor (mTERF) family consists of a group of nucleic acid binding proteins with so-called mTERF repeats of ~ 31 amino acids forming three helices [1, 2]. Similarity searches and phylogenetic analysis demonstrated that the mTERF family exists only in eukaryotes except for fungi [3]. All these proteins are predicted to localize to mitochondria and/or chloroplasts. Mammal genomes encode only four mTERFs (MTERF1–4) [4], while higher plants harbor approximately 30 members [5]. The mammalian mTERFs regulate mitochondrial gene expression. Human MTERF1, the first identified mTERF, functions in terminating L-strand transcription at the 16S rRNA/leucyl-tRNA boundary [6], transcription activation [7, 8], and DNA replication [9], followed by the discoveries of the roles of MTERF2 in restraining replication fork progression [10], MTERF3 in transcription suppression, replication [11], and ribosomal biogenesis [12], and MTERF4 in transcription activation [13] and ribosomal biogenesis [14]. Plant mTERFs, by contrast, are barely understood for their roles in the regulation of organellar gene expression. Of the 35 mTERFs in Arabidopsis, 11 are chloroplast-localized [15]. SOLDAT10, the first mTERF characterized in higher plants, participates in stress acclimation response and affects the abundance of 16S and 23S rRNA and ClpP protease mRNA [16] in chloroplasts. BSM (RUG2) is targeted to both mitochondria and chloroplasts and is required for the maintenance of the constant accumulation of transcripts in these two organelles [15, 17], which includes splicing of the *clpP* group IIA intron [15]. Two comparative analyses of two other chloroplastic mTERFs, mTERF5 (MDA1) and mTERF9 (TWIRT1), indicated a functional relationship between them, that they share some common targets in gene expression regulation, both respond to salt and osmotic stresses, and both are functionally related to the plastid-encoded plastid RNA polymerase (PEP) [18]. Also, TWIRT1 is likely required for plastid ribosomal stability and/or assembl y[19], and MDA1 positively regulates *psbEFLJ* transcription as a transcriptional pausing factor [20], stimulates *psbE* and *ndhA* transcription, and promotes the stabilization of the 5′-ends of processed *psbE* and *ndhA* mRNA [21]. A recent report further found that transcription termination of *psbJ* in the *psbEFLJ* polycistron also involved another PEP-associated mTERF, *mTERF8/pTAC15*, which specifically binds to the 3′ terminal region of *psbJ* [22]. In addition to the studies on chloroplastic mTERFs, two mitochondrial mTERFs in *Arabidopsis*, mTERF18 (SHOT1) and mTERF15, were found to interfere in retrograde signaling for heat tolerance [23], and splicing of the *nad2* intron-3 [24], respectively. Meanwhile, a few studies provided some clues to the action modes of plant mTERFs. Zm-mTERF4, the BSM ortholog in maize, directly binds the group II introns in certain chloroplastic transcripts and interacts with some of the known chloroplastic splicing factors, thus promoting the splicing of such transcripts, including *trnI*-*GAU, trnA*-*UGC*, and *rpl2* [25]. Later, two studies of *Arabidopsis* mTERF6 demonstrated its DNA-binding activity in vivo, which is required for the transcription termination at a specific site in *trnI*-*GAU* and at the 3′-end of *rpoA* polycistron in chloroplasts [26, 27]. Recently, mitochondrial *ZmSmk3* was found involved in the splicing of *nad4* intron 1 and *nad1* intron 4 in maize [28], and Arabidopsis mTERF9 was shown to promote chloroplast ribosome assembly and translation by interacting with 16S and 23S rRNAs [29]. Despite these advances, the molecular mechanism by which plant mTERFs regulate organellar gene expression is still far from full understanding, and it is not clear if mTERFs involve in processing organellar polycistronic transcripts. Moreover, little information has been provided so far for the impact of mTERFs on chloroplast and mitochondrion development in rice (*O. sativa* L.), a model crop species.

Derived from a cyanobacterial ancestor, the chloroplast holds many genes organized in gene clusters. Chloroplast mRNA maturation includes multiple steps, which are precursor transcription, 5′ and 3′ end processing, intercistronic cleavage, 5′ and 3′ end maturation and editing, and intron removal [30]. At least two distinct RNA polymerases, PEP and the nucleus-encoded RNA polymerase (NEP), are responsible for plastid gene transcription during all phases of chloroplast development and in non-green plastid types [31]. The gene encoding the α subunit of PEP, *rpoA*, is clustered with multiple ribosomal protein-encoding genes in the *rpoA* operon, while the genes encoding the β, β′ and β″ subunits of PEP, *rpoB*, *rpoC1*, and *rpoC2*, respectively, form a separate operon. Both of the *rpoA* and *rpoB* operons are transcribed by NEP [31–33] On the other hand, photosynthetic genes, such as *psbA*, *psbD*, and *psaB*, are PEP-dependent. The association between PEP and the promoter regions of most of these genes is significantly increased in the light [34]. For example, a light-responsive promoter was identified between *psbI* and *psbD* in the *psbK*-*psbI*-*psbD*-*psbC* operon, which accounts for the transcription of the dicistronic *psbD*-*psbC*. Two other standard PEP promoters residing upstream of *psbK* and the light-responsive promoter, respectively, otherwise produce five different overlapping transcripts including *psbK*-*psbI*-*psbD*-*psbC*, *psbK*-*psbI*, and *psbD*-*psbC* [35]. The light-induced *psbD*-*psbC*, which was undetectable in the dark, was abundantly accumulated in green rice seedlings [35].

Here we described the effects of a rice mTERF, V14, on appropriate intercistronic cleavage of the polycistronic *rpoB*-*rpoC1*-*rpoC2* precursor in chloroplasts and accumulations of certain photosystem proteins, for example PsbD, during early stage of seedling leaf development. Intriguingly, this regulation pattern is growth stage-specific and temperature-sensitive. We thus suggest a role of V14 in chloroplast development and adaptation to temperature.

## Results

### V14 is a chloroplastic protein critical to early stage of leaf development

**Fig. 1:**
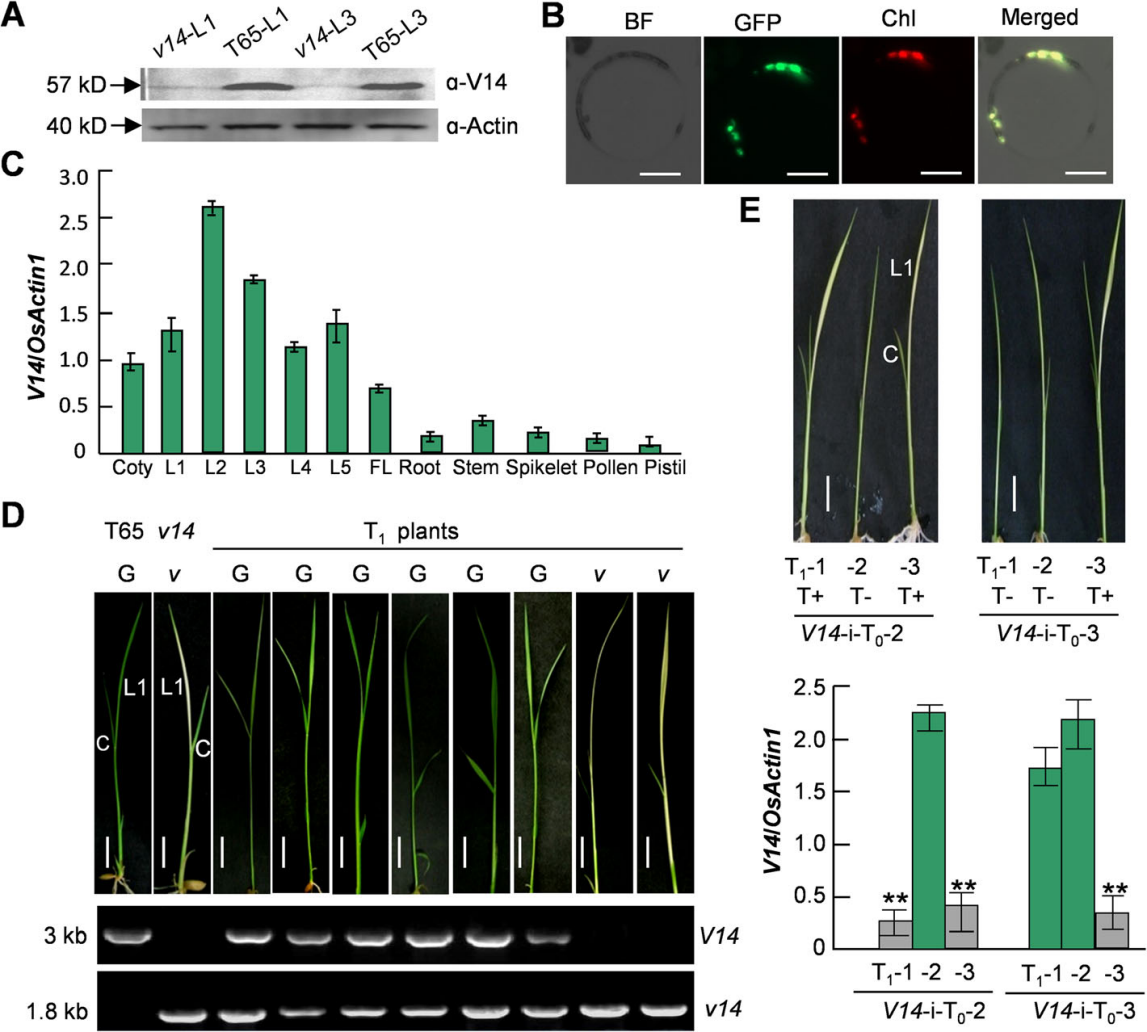
V14 is critical to leaf development in the early stages of seedling. **A** V14 deficiency in the *v14* mutant demonstrated by immunoblotting analysis of the first and third leaves of *v14*. **B** Chloroplast localization of V14 indicated by the green fluorescence expressed by V14-fused EGFP in chloroplasts. BF, bright field; Chl, autofluorescence of chlorophyll (red); Bar = 10 μM. **C** Expression of *V14* in different organs and at different stages of development. Coty, cotyledon; L1-L5, the first to the fifth true leaf; FL, flag leaf. **D** Complementation of the *v14* mutant confirmed by phenotypic analysis and *V14-* and *v14*-specific PCR in T_1_ plants. G, green; *v*, virescent. The un-cropped gel image is provided in Additional file 1 B. Bar = 0.5 cm (**E**) Reproduction of the *v14*-like phenotype in the T_1_ generation of two independent *V14*-RNAi lines, which is confirmed by phenotypic analyses and the gene expression analysis of *V14* by qRT-PCR. Bar = 0.5 cm. The significant difference between T+ (with the transgene) and T- (without the transgene) plants was analyzed by student’s *t* test (*n* = 3). *, *P* < 0.05; **, *P* < 0.01. (**A**) and (**B**), The data presented here are the representative images of three independent experiments. (**C**) and (**E**), The relative expression levels shown here are the averages of three independent experiments

The *V14* locus was previously mapped as a 162-kb region on chromosome 7, using a stage-conditional *virescent*-*14* (*v14*) mutant of Taichung 65 (T65), a *japonica* cultivar [36]. This mutant develops albinism in the first two true leaves at 25 °C and returns green thereafter [30]. Subsequent fine mapping narrowed down the *V14* locus to a 30-kb region containing two protein-coding genes (Additional file 1). One of which, *Os07g0583200*, had a 1283-bp deletion in the promoter and 5′ untranslated/coding regions (− 1245 ~ + 38) in the mutant [36]. Indeed, deficiencies of *Os07g0583200* mRNA [36] and its protein product (Fig. 1A) were observed in *v14*. This gene encodes a putative chloroplastic protein (Refseq, https://www.ncbi.nlm.nih.gov), and its chloroplast localization was verified in rice leaf protoplasts expressing a *Os07g0583200*-fused *eGFP* construct (Fig. 1B). These data suggested that *Os07g0583200* was a strong candidate for the *V14* locus. Transcripts of this gene were observed in all analyzed stages of leaves (Fig. 1C), and their products bear seven consecutive mTERF repeats at the C-terminus [36], which is annotated by Refseq as a rice MTERF9. We further performed transgenic complementation and RNA interference (RNAi) to confirm *Os07g0583200* represents the *V14* locus. Successful complementation of the albinism was achieved in those transgenic *v14*-T_1_ plants carrying an *Os07g0583200*-containing fragment with its native promoter (Fig. 1D). Simultaneously, the *v14*-like phenotype was reproduced in the RNAi plants manifesting down-regulated *Os07g0583200* expression (Fig. 1E). We therefore assigned *V14* to this gene thereafter.

### V14 sustains functional chloroplasts via posttranscriptional precursor cleavage

**Fig. 2:**
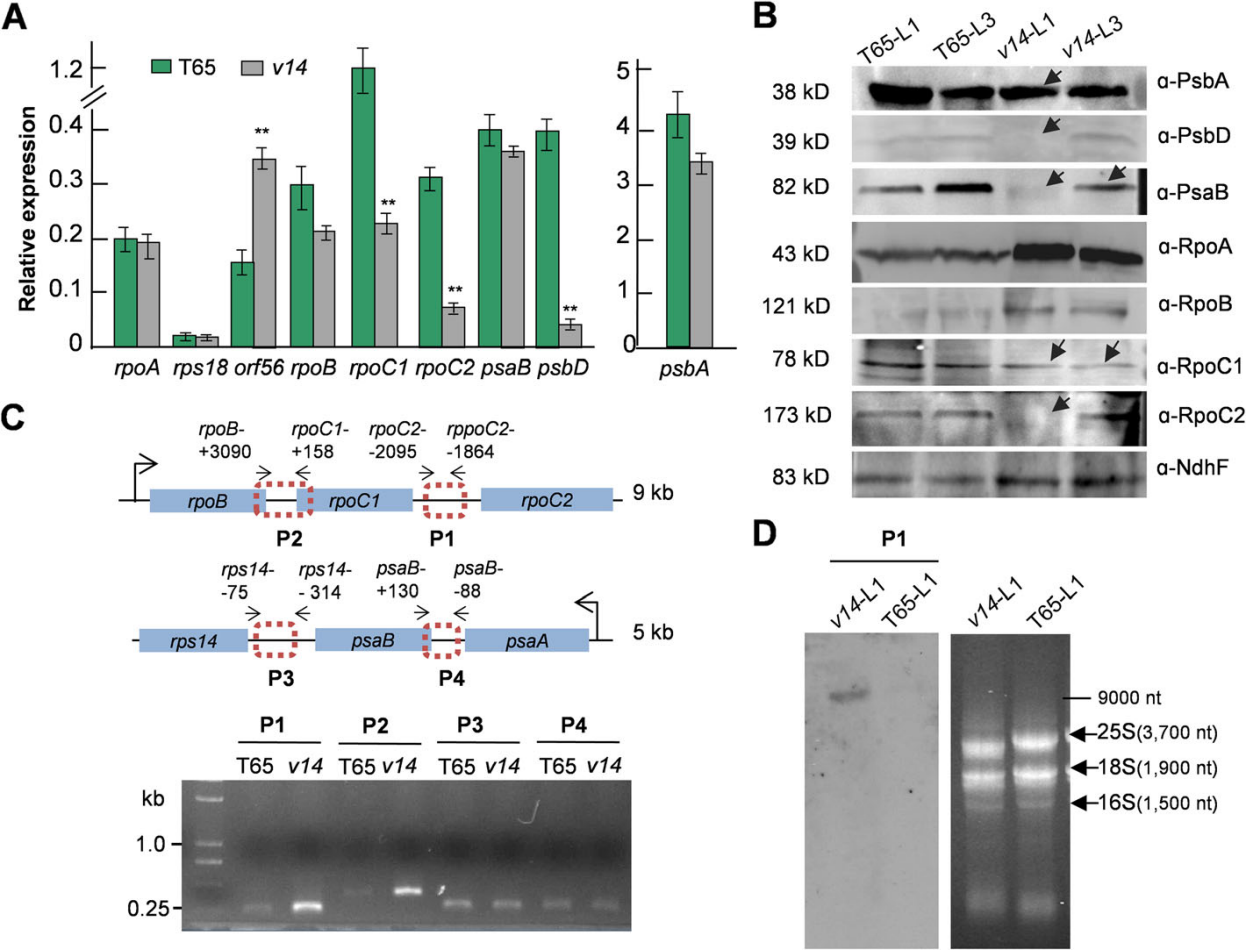
V14 affects the intercistronic cleavage of the *rpoB*-*rpoC1*-*rpoC2* precursor, *psbD* transcript abundance, and PsbA and PsaB accumulations during early seedling development. **A** Expression analysis of the chloroplastic genes by qRT-PCR in seedlings (L1) grown at 25 °C. The stably expressed chloroplastic gene *psbE* was used for normalization. Only the genes showing significant changes in *v14* and some of the “unchanged” genes are shown here. The significance compared to T65 was analyzed by *t* test (*n =* 3). *, *P* < 0.05; **, *P* < 0.01. **B** Immunoblotting analysis of chloroplastic proteins in L1 and L3 with commercially available antibodies. Arrows indicate reduced or no accumulation of the proteins. A stably-expressed plastid protein NdhF was used as an internal control. **C** Aberrant accumulation of the precursor transcripts covering the two intercistronic regions (P1 and P2) of *rpoB*-*rpoC1*-*rpoC2* in L1 (grown at 25 °C) of *v14*, as indicated by semi-quantitative RT-PCR (31 cycles). The cleavage assays for the two intercistronic regions (P3 and P4) of *psaA*-*psaB*-*rps14* were used as the control. The positions of the primers are designated relative to the start codon of the ORFs downstream or those where they are located. **D** Significantly higher accumulation of the unprocessed precursor *rpoB*-*rpoC1*-*rpoC2* in L1 of *v14* as compared to T65, as shown by Northern hybridization. L1 and L3, the first and the third true leaf, respectively. (**B**), (**C**), and (**D**), The images presented here are the representatives of three biological repeats

Our prior knowledge of *v14* indicates an arrest of chloroplast development in the first two true leaves, as is evident from the absence of mature thylakoids and starch grains [36]. We thus posited transcription deficiency residing in the chloroplasts of the chlorotic leaves. To determine the chloroplastic genes affected, we first used qRT-PCR to assess the difference between *v14* and T65 in transcript abundance of all 62 chloroplastic genes in the first true leaves. The results revealed significant gene expression changes (2 fold minimum) in *v14* as compared to the wild-type T65, which involved two genes encoding the β′-, and β”-subunits of PEP, *rpoC1* and *rpoC2*, a photosynthetic gene *psbD*, and a rice-exclusive gene *orf56* [37, 38] encoding a truncated NdhH [39] (Fig. 2A). The transcripts *rpoC1*, *rpoC2*, and *psbD* were considerably down-regulated in *v14*, whilst *orf56* was up-regulated (Fig. 2A). Further semi-quantitative RT-PCR analysis of the overlapping region across the coding sequences of *psbD* and *psbC* revealed its absence in *v14* (Additional file 2A), confirming the significant reduction in *psbD* mRNA abundance. Given the important roles of the nucleus-encoded sigma factors in PEP activation, the expression of all five sigma factors was also analyzed by qRT-PCR. The results indicated that none of them were affected by V14 deficiency (Additional file 2B). Subsequent immunoblotting analysis confirmed the protein deficiency of RpoC1, RpoC2, and PsbD in *v14* (Fig. 2B, Additional file 2C). In addition, the protein levels of some other photosynthetic proteins were assessed with commercially available antibodies. We found that two other photosystem proteins PsbA and PsaB also decreased in *v14* (Fig. 2B, Additional file 2C), even though their transcript abundance was unchanged (Fig. 2A). In contrast, RpoA and RpoB were more abundant in *v14* than in T65 (Fig. 2B, Additional file 2C). This might be attributed to the feedback inhibition of translation that has been observed in bacteria [40].

We next analyzed how V14 influences mRNA levels in chloroplasts. We noted that the V14 target genes are organized in co-transcribed gene clusters with other non-targeted genes. In light of the role of maize Zm-mTERF4 in intron splicing [25], we hypothesized that V14 may intervene in either precursor cleavage or RNA stabilization. To explore the behavior of V14, the abundance of the two intercistronic regions (P1 and P2) from the polycistronic *rpoB*-*rpoC1*-*rpoC2* precursor (Fig. 2C) were analyzed by semi-quantitative RT-PCR in the first two true leaves of *v14* and T65, using the two intercistronic regions (P3 and P4) from the polycistronic *psaA*-*psaB*-*rps14* precursor (Fig. 2C) as controls. We found that the chlorotic leaves boasted higher amounts of the unprocessed transcripts including the *rpoB*-*rpoC1* and/or *rpoC1*-*rpoC2* regions as compared to the wild-type (Fig. 2C, Additional file 2D). No significant changes were observed for the P3 and P4 regions in *v14* (Fig. 2C, Additional file 2D). Using P1 as a probe for Northern hybridization, we also detected a ~ 9-kb precursor transcript containing *rpoB*, *rpoC1*, and *rpoC2* only in *v14* (Fig. 2D).

Taken together, these data reflect the effects of V14 on the appropriate intercistronic cleavage of polycistronic *rpoB*-*rpoC1*-*rpoC2* and *psbD* mRNA, PsbA, and PsaB accumulations. Given that light regulates expression of the PEP components in the first phase of photosynthesis establishment and *psbD* mRNA, PsbA, and PsaB abundance [41–44], V14 may play a key role in light signaling through chloroplast development.

### The albinism phenotype of *v14* is temperature-dependent

**Fig. 3:**
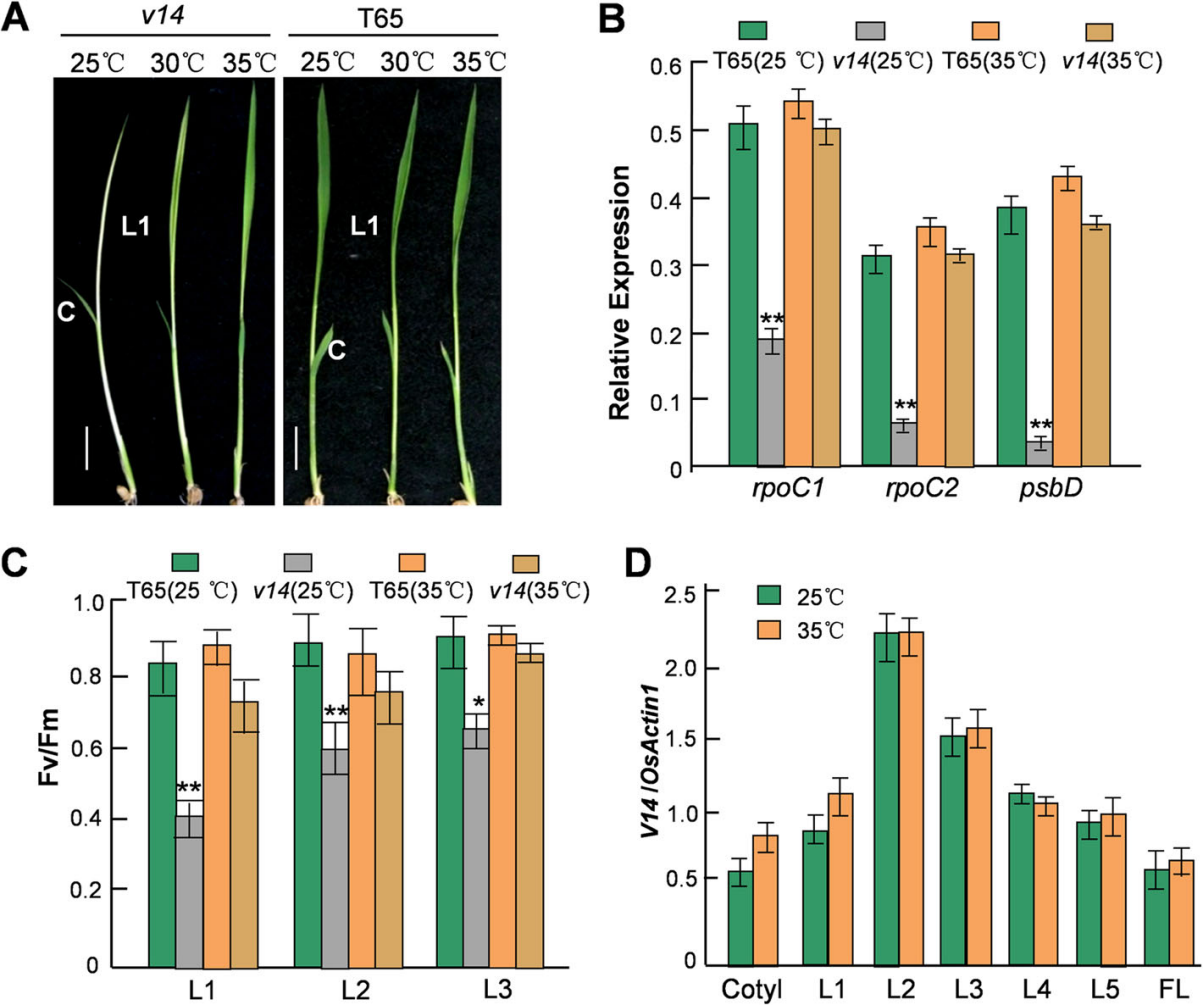
*v14*-induced defective posttranscriptional regulation and *psbD* mRNA reduction is reversed by high temperatures. **A** The chlorotic leaves of *v14* returned green at 30 °C and 35 °C. Bar = 0.5 cm. **B** qRT-PCR analysis showed the expression recuperation of *rpoC1*, *rpoC2*, and *psbD* in L1 of *v14* at 35 °C. **C** The maximum photosynthetic quantum yields represented as the Fv/Fm ratios recorded in L1, L2, and L3 of *v14* confirmed its recovery of chloroplast function at 35 °C. (**B**) and (**C**), The significance compared to T65 grown at 25 °C was analyzed by *t* test (*n =* 3) . *, *P* < 0.05; **, *P* < 0.01. **D** qRT-PCR showing that *V14* is continuously expressed in leaves and unresponsive to high temperature. L1-L3, the first to the third true leaf. The relative expression levels shown here are the averages of three independent experiments

Similar to the three temperature-conditional rice virescents reported previously [45], *v14* developed green leaves at permissive temperatures, 30 °C and 35 °C (Fig. 3A). To ascertain this full recovery at high temperatures, we first evaluated the expression levels of *rpoC1*, *rpoC2*, and *psbD* in the first true leaves of the *v14* plants grown at 25 °C and 35 °C. The RT-PCR analysis showed that the abundance of these transcripts at 35 °C could reach a level comparable to their counterparts in T65 (Fig. 3B). Indeed, the appropriate cleavages of the two intercistronic regions of *rpoB*-*rpoC1*-*rpoC2* were retrieved in these green-recovered *v14* plants (Additional file 3). We further assessed chloroplast function in the *v14* plants grown at 35 °C by gauging the Fv/Fm ratio (the maximum photosynthetic quantum yield), a measurement representing Photosystem II efficiency, in the first, second, and third true leaves. In agreement with the expression recuperation of *rpoC1*, *rpoC2*, and *psbD*, the Fv/Fm values recorded in the 35 °C-growing *v14* plants showed no significant difference from those in the T65s grown either at 25 °C or 35 °C (Fig. 3C). By contrast, the 25 °C-growing *v14* plants still could not fully retrieve the power of photosynthesis in their third leaves even though they returned green (Fig. 3C). Interestingly, *V14* mRNA expression in T65 was neither heat-sensitive nor stage-specific, albeit relatively high in the second true leaf (Figs. 1C, 3D).

**Fig. 4:**
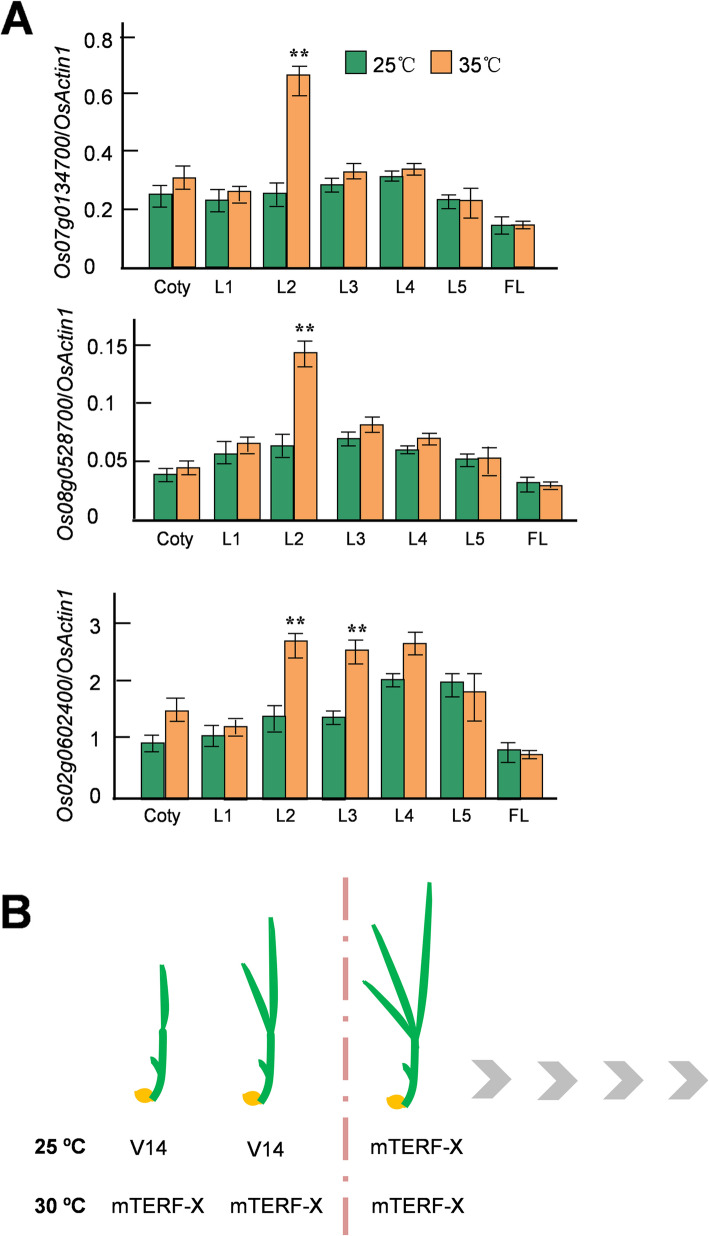
**A** Three rice mTERF genes are heat-inducible, as indicated by qRT-PCR. The significant difference in expression between growth at 35 °C and 25 °C was analyzed by *t* test (*n =* 3). *, *P* < 0.05; **, *P* < 0.01. **B** The effects of V14 on chloroplast development are stage-specific and themo-sensitive

We thus postulated that there may be unknown V14 parallel factor(s) acting at high temperatures while V14 is inactive. Our similarity searches on NCBI identified 30 other V14-homologs in rice (Additional file 4). Expression analysis of these genes in 25 °C- and 35 °C-grown T65s showed that three of them, *Os07g0134700*, *Os08g0528700*, and *Os02g0602400*, were significantly up-regulated at 35 °C in the second true leaf where *V14* expression reaches its peak (Fig. 4A, Additional files 5, 6 and 7). *Os07g0134700* and *Os02g0602400* are predicted to encode chloroplastic mTERFs (annotated as rice MTERF2 and MTERF5 homologs, respectively, by Refseq), while *Os08g0528700* encodes an unannotated mTERF-like protein.

## Discussion

The mTERF family earned its name from its founding member, human MTERF1 [6], as a group of transcription termination factors 31 years ago, but more molecular functions have since been linked to it, such as transcription initiation, DNA replication, and intron splicing. Our data presented here extend the understanding of the molecular functions of mTERFs, which may regulate intercistronic cleavage of polycistronic precursors. We showed that V14 is required for the appropriate intercistronic cleavage of *rpoB*-*rpoC1*-*rpoC2* precursor, thus regulating the abundance of mature *rpoC1* and *rpoC2* mRNAs that encode two core subunits of PEP. The expression of *rpoB*, however, is not targeted by V14. Since *rpoB* is co-transcribed with *rpoC1* and *rpoC2* by NEP [31–33], this result indicates that V14 specifically regulates precursor processing but not precursor transcription for the *rpoB*-*rpoC1*-*rpoC2* operon. Despite reduced expression of mature *rpoC1* and *rpoC2* mRNAs in *v14*, expression of the PEP-dependent genes is unaffected except for *psbD*, implicating that low levels of PEP in the proplastids can maintain the expression of most of these genes. This observation is consistent with the developmental and gene-specific regulation of PEP transcription proposed in wheat seedlings [43]. In developing chloroplasts the light-independent PEP functions in the dark as well as in the light for the PEP-dependent genes including *psbA*, *psbC*, *psbE*, and *rrn16*, except for *psbD*, whilst the light-dependent PEP selectively transcribes *psbA* and *psbD* in mature chloroplasts [43]. A light-responsive promoter producing a precursor including *psbD* and *psbC* has also been identified in the *psbK*-*psbI*-*psbD*-*psbC* gene cluster [46]. Considering *psbC* showed no expression change in *v14* while being co-transcribed with *psbD*, we speculated that V14 may be crucial for *psbD* mRNA stability in a light-dependent way. We also noted that two other photosystem proteins PsbA and PsaB also decreased because of the V14 deficiency even without alterations in their transcript abundance, which can be explained by the facts that translation and stability of proteins encoded by *psbA* and *psaB* are light-dependent during chloroplast development [41]. Furthermore, a light response model established in Arabidopsis indicates that light signals precede plastid signals, where the first phase of photosynthesis establishment relies on light and triggers changes that will initiate chloroplast development, and more importantly initiates expression of the PEP components [47]. This model supports the impaired processing of *rpoB*-*rpoC1*-*rpoC2* precursor and reduced accumulations of *psbD*, PsbA, and PsaB observed in the chlorotic leaves of *v14*, suggesting that V14 is essential for light signaling during chloroplast development. V14 might not act directly on its molecular targets, such as the *rpoB*-*rpoC1*-*rpoC2* precursor, but interfere in a key step during chloroplast development that introduces these changes, which needs further investigations.

We showed that *v14* was rescued by higher temperatures, and that the defective intercistronic cleavage of the *rpoB*-*rpoC1*-*rpoC2* precursor is temperature-dependent (Fig. 3B, Additional file 3). However, the expression of *V14* per se in wild-type is not temperature-sensitive (Fig. 3D). Considering V14 might act via phytohormone-mediated thermosensory pathways [48, 49], we examined the response of *v14* seedlings to various phytohormone treatments (Additional file 8). However, none of such treatments could restore the albinism observed at 25 °C, suggesting that *V14* is irresponsive to phytohormones. We further identified three mTERF genes showing a similar temporal expression pattern to *V14* that were significantly up-regulated by high temperatures in the second leaf (Fig. 4A), suggesting that these genes may be potential candidates for the unknown V14 parallel(s) whose functions compensate for the V14 deficiency at high temperatures. These data support the notion that plants have evolved functionally redundant members of gene families, by which certain members can be replaced by some other members in a conditional manner.

## Conclusion

V14 is an essential transcriptional and translational regulator in chloroplasts supporting chloroplast biogenesis in the first two true leaves. While *V14* expression is neither stage-specific nor thermo-sensitive, the V14-mediated regulation is stringently modulated by developmental stages and temperature (Fig. 4B). The roles of V14 in chloroplast development are just beginning to emerge. Further studies are needed to dissect the molecular functions of V14 in intercistronic cleavage, mRNA stability, and translational/posttranslational regulation, and to define how such regulations respond to temperature, thus helping to understand the contributions of organellar gene expression to photosynthesis establishment and temperature acclimation.

## Methods

### Plant materials and treatment

The seeds of T65 were obtained from Dr. Chuxiong Zhuang’s lab at South China Agricultural University. The seedlings were grown in growth chambers under 16-h light/8-h dark cycles at 25 °C. Details for the positional mapping of the *V14* locus were provided in a previous study [36]. For the temperature treatments, the seedlings were grown under 16-h light/8-h dark cycles at 25 °C, 30 °C or 35 °C.

### Intracellular localization of eGFP fusions

For eGFP visualization, a cDNA fragment of *V14* was obtained from T65 using the primers V14-xho5 and V14-spe3 (Additional file 9), and fused with the coding sequence of *eGFP* in a pUC18-based vector to create the construct *P35S*::*V14*-*eGFP*. The construct was then transiently transformed into rice leaf protoplasts following the protocol described previously [50]. The images were collected in the 500- to 550-nm (eGFP fluorescence), and 670- to 750-nm (chlorophyll autofluorescence) ranges with a laser confocal scanning microscope fitted with a 40 × water immersion objective (7 DUO; Zeiss).

### Genetic transformation

For complementation test of the *v14* mutant, a genomic fragment containing the promoter, gene body, and a 914-bp 3′ UTR region was amplified from T65 genomic DNA using the primers V14P5 and V14P3 (Additional file 9, and cloned into a plant expression vector pCAMBIA 1380 with Gibson Assembly® Master Mix (New England Biolabs). The success of the complementation was confirmed by phenotypic analysis and PCR using the *V14*-specific primers as described in a previous study [36]. For generation of the *V14*-RNAi plants, a cDNA fragment was obtained from T65 using two primer pairs (Additional file 9), V14i-5-1 and V14i-3-1, and V14i-5-2 and V14i-3-2, and cloned into a plant expression vector pCAMBIA 1301 with Gibson Assembly® Master Mix. The complementation construct was introduced into *v14*, and the RNAi one was transferred into T65, by Agrobacterium-mediated transformation.

### Chloroplast isolation

Chloroplasts were prepared as previously published [51]**.** In brief, 10 g of fresh seedling leaves were frozen in liquid nitrogen and gently ground into fine powder. The powder was then suspended in 100 ml of Medium A (50 mM HEPES-KOH pH 8.0, 330 mM sorbitol, 2 mM EDTA-Na_2_, 5 mM ascorbic acid, 5 mM cysteine, 0.05% BSA) and the suspension was filtered through two layers of gauze and then two layers of Miracloth (Merck). This filtrate was subjected to centrifugation (1300×g, 4 °C, 5 min) to collect chloroplast pellets followed by sucrose density gradient centrifugation (30, 40, 55% sucrose density gradient in Medium B, 30000×g, 4 °C, 1 h) using the pellets suspended in 200 μl of Medium B (50 mM HEPES-KOH pH 8.0, 330 mM sorbitol, 2 mM EDTA-Na_2_). The green band at the 30 and 40% sucrose interface was collected and rinsed twice with 75 mL of Medium B through centrifugation (2000×g, 4 °C, 15 min). Finally, the pellets were resuspended in 50 μl of TRIzol™ Reagent (ThermoFisher Scientific) for RNA extraction.

### Nucleic acid extraction, qRT-PCR, northern blot and immunoblotting

Genomic DNA was isolated from leaves with a DNeasy Plant Mini Kit (Qiagen). Total RNA was extracted from leaves or chloroplasts following the instruction of TRIzol® Reagent. DNase I (Invitrogen) digestion was applied prior to reverse transcription. For assessment of the abundance of nuclear transcripts, Oligo (dT)_20_ (50 μM) was used for the synthesis of first-strand cDNA from total RNA extracted from leaves. For assessment of the amounts of all chloroplastic transcripts, Random Hexamers (50 ng/μL) was used for the synthesis of first-strand cDNA from total RNA extracted from chloroplasts. The primers for the nucleus-encoded sigma factors were shown in Additional file 9. All the primer sequences for detecting chloroplastic transcripts were listed in Additional file 10, except for those for amplifying the intercistronic regions (P1-P4), the overlapping region across *psbD* and *psbC*, and a region downstream of *psbC* (Additional file 2 A), which were given in Additional file 9. Northern blot analysis was performed using total RNA as previously published [52]. The probe P1 was labeled with 0.01 μM digoxigenin (DIG)-deoxyuridine triphosphate by PCR. To extract proteins, seedling leaves were homogenized in 2 × SDS sample buffer (62.5 mm Tris-HCl, pH 6.8, 20% [v/v] glycerol, 4% [w/v] SDS, 100 mm dithiothreitol, and 0.05% [w/v] Bromophenol Blue), incubated at 95 °C for 5 min, and centrifuged at the maximum speed for 20 min. The samples were quantified and subjected to SDS-PAGE (12%) followed by wet transfer to PVDF membranes (Millipore). The membranes were then incubated with the antibodies against V14, RpoA, RpoB, RpoC1, RpoC2, PsbA, PsbD, PsaB, or NdhF. All these antibodies were obtained from BGI, except for the V14 antibody, which was developed by Abmart using a synthetic peptide EGRQPKTRDRCD as the immunogen. All the protein levels were normalized to NdhF by ImageJ, which is shown in Additional file 2C.

### Chlorophyll fluorescence analysis

The experiments were performed following the protocol published previously [53] with some minor modifications. Six plants for each group were dark-adapted for 20 min before taking measurements with a PAM fluorometer (Walz). All measurements were taken at the same time during the day. A saturating pulse of radiation (2700 μmol m^− 2^ s^− 1^) was applied to record the maximum fluorescence yield (Fm), and a weak modulating radiation (0.5 μmol m^− 2^ s ^− 1^) was used to measure the minimum fluorescence yield (F_0_). The maximum photosynthetic quantum yield was then calculated as Fv (variable fluorescence yield)/Fm = (Fm-F_0_)/Fm.

## Supplementary Information

**Additional file 1. **(A) Fine mapping of the *V14* locus on chromosome 7. The dashed line represents the genomic deletion in the promoter and 5’untranslated and coding regions (− 1245 ~ + 38) in the *v14* mutant. Arrows indicate primers for detecting the endogenous and transgenic fragments of *V14* and *v14* as shown in Fig. 1D. (B) Complementation of the *v14* mutant confirmed by *V14-* and *v14*-specific PCR in T_1_ plants.

**Additional file 2. **Analysis of the *psbD*-containing transcripts by semi-quantitative RT-PCR (31 cycles) (A), expression analysis of the rice sigma factors by qRT-PCR (B), quantification of the immunoblotting analysis shown in Fig. 2B by ImageJ (C), and analysis of the two intercistronic regions (P1 and P2) of *rpoB*-*rpoC1*-*rpoC2* in the second true leaves (grown at 25 °C) of *v14* by semi-quantitative RT-PCR (31 cycles) (D). (A), The positions of the primers are designated relative to the start codon of the ORFs where they are located. (B), The significance compared to T65 was analyzed by *t* test (*n* = 3). (C), All the protein levels were normalized to NdhF. (D), The two intercistronic regions (P3 and P4) of *psaA*-*psaB*-*rps14* were used as the control. (A) and (D), The images presented here are the representatives of three biological repeats.

**Additional file 3. **High temperature (35 °C) rescued the cleavage of the two intercistronic regions (P1 and P2) of the *rpoB*-*rpoC1*-*rpoC2* precursor in L1 of *v14*. The semi-quantitative RT-PCR was carried out by 31 cycles. L1, the first leaf. P3 and P4 are the two spacer regions in the *psaA*-*psaB*-*rps14* operon shown in Fig. 2C. The image presented here is the representative of three biological repeats.

**Additional file 4.** Phylogenetic analysis of V14 in rice. The neighbor-joining tree was built on protein sequences using the software PHYLIP (version 3.66) and visualized with the software TreeView and MEGA5. Arrows indicate the three genes with temperature-sensitive expression in the second leaf (Fig. 4).

**Additional file 5. **Gene expression profiles of the other 27 *V14*-homologous genes at different stages of leaf development at 25 °C and 35 °C. The relative expression levels shown here are the averages of three independent experiments.

**Additional file 6. **Gene expression profiles of the other 27 *V14*-homologous genes at different stages of leaf development at 25 °C and 35 °C. The relative expression levels shown here are the averages of three independent experiments.

**Additional file 7. **Gene expression profiles of the other 27 *V14*-homologous genes at different stages of leaf development at 25 °C and 35 °C. The relative expression levels shown here are the averages of three independent experiments.

**Additional file 8 ***v14* seedlings on various phytophormone treatments at 25 °C. Bar = 0.5 cm; L1, the first true leaf; L2, the second true leaf.

**Additional file 9.** Primer sequences for complementation, RNAi, and the assessment of the processing intermediates.

**Additional file 10.** Primer sequences for the mRNA expression profiling of the chloroplastic gene transcripts.

**Additional file 11.** The un-cropped blot images of Figs. 1A, 2B, and D.

**Additional file 12.** The original real-time PCR data for Figs. 2A, 3B, and 4A.

## Data Availability

The sequences of *V14* and the mutant *v14* have been deposited in NCBI under the submission IDs MZ299153 and MZ299154, respectively.

## Abbreviations

mTERF: Mitochondrial transcription termination factor
PEP: The plastid-encoded plastid RNA polymerase
NEP: The nucleus-encoded RNA polymerase
Fv: Variable fluorescence yield
Fm: The maximum fluorescence yield
F_0_: The minimum fluorescence yield
NCBI: National Center for Biotechnology Information
