# Supplementary figures and images for: A rice mTERF protein V14 sustains photosynthesis establishment and temperature acclimation in early seedling leaves
Source: BMC Plant Biol. 2021 Sep 6;21:406. doi: 10.1186/s12870-021-03192-2 (PMC8420055; doi:10.1186/s12870-021-03192-2)

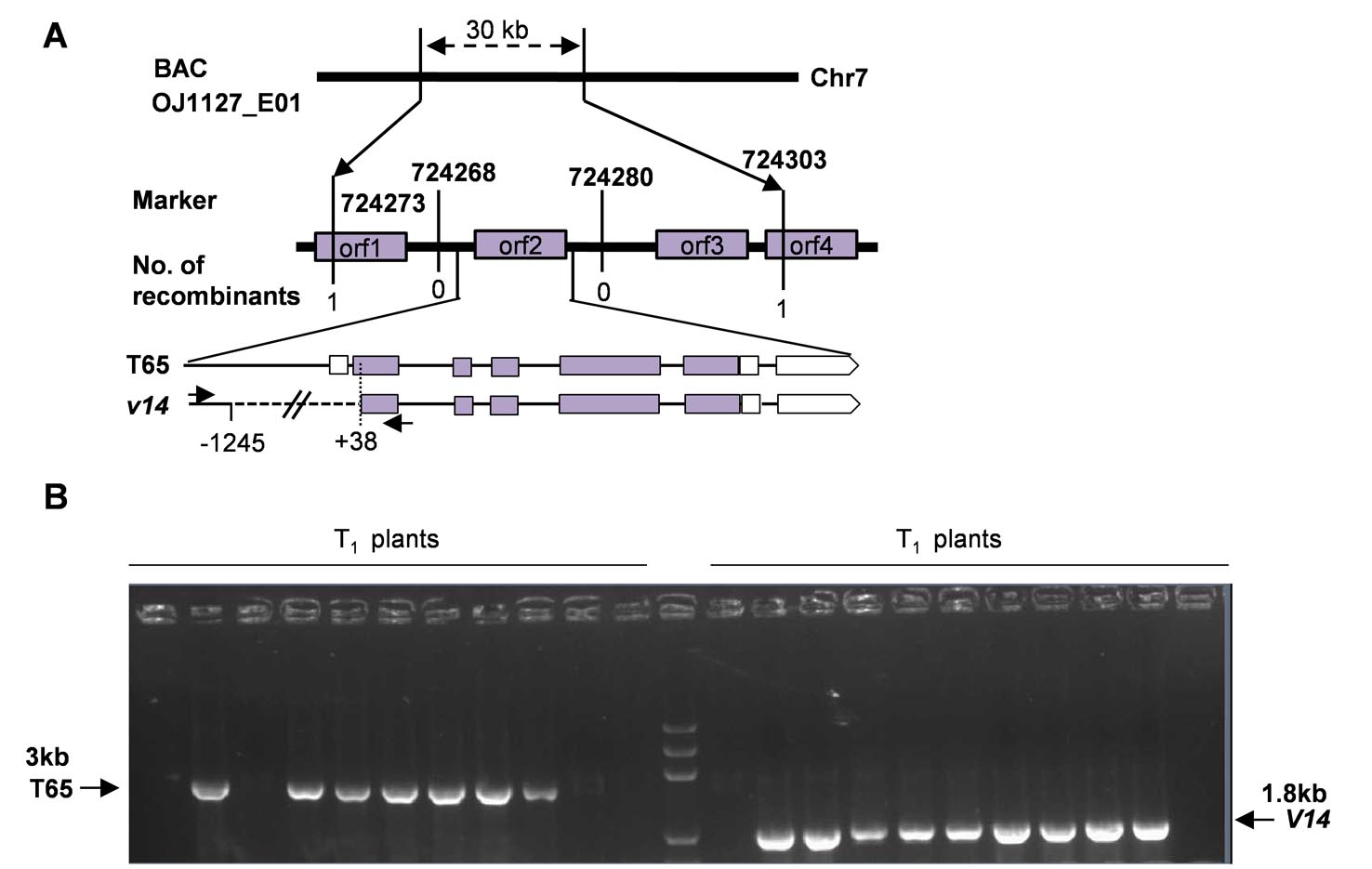

Supplement: Supplementary file 1 — Additional file 1. (A) Fine mapping of the V14 locus on chromosome 7. The dashed line represents the genomic deletion in the promoter and 5’untranslated and coding regions (− 1245 ~ + 38) in the v14 mutant. Arrows indicate primers for detecting the endogenous and transgenic fragments of V14 and v14 as shown in Fig. 1D. (B) Complementation of the v14 mutant confirmed by V14- and v14-specific PCR in T1 plants. [file 12870_2021_3192_MOESM1_ESM.jpg]

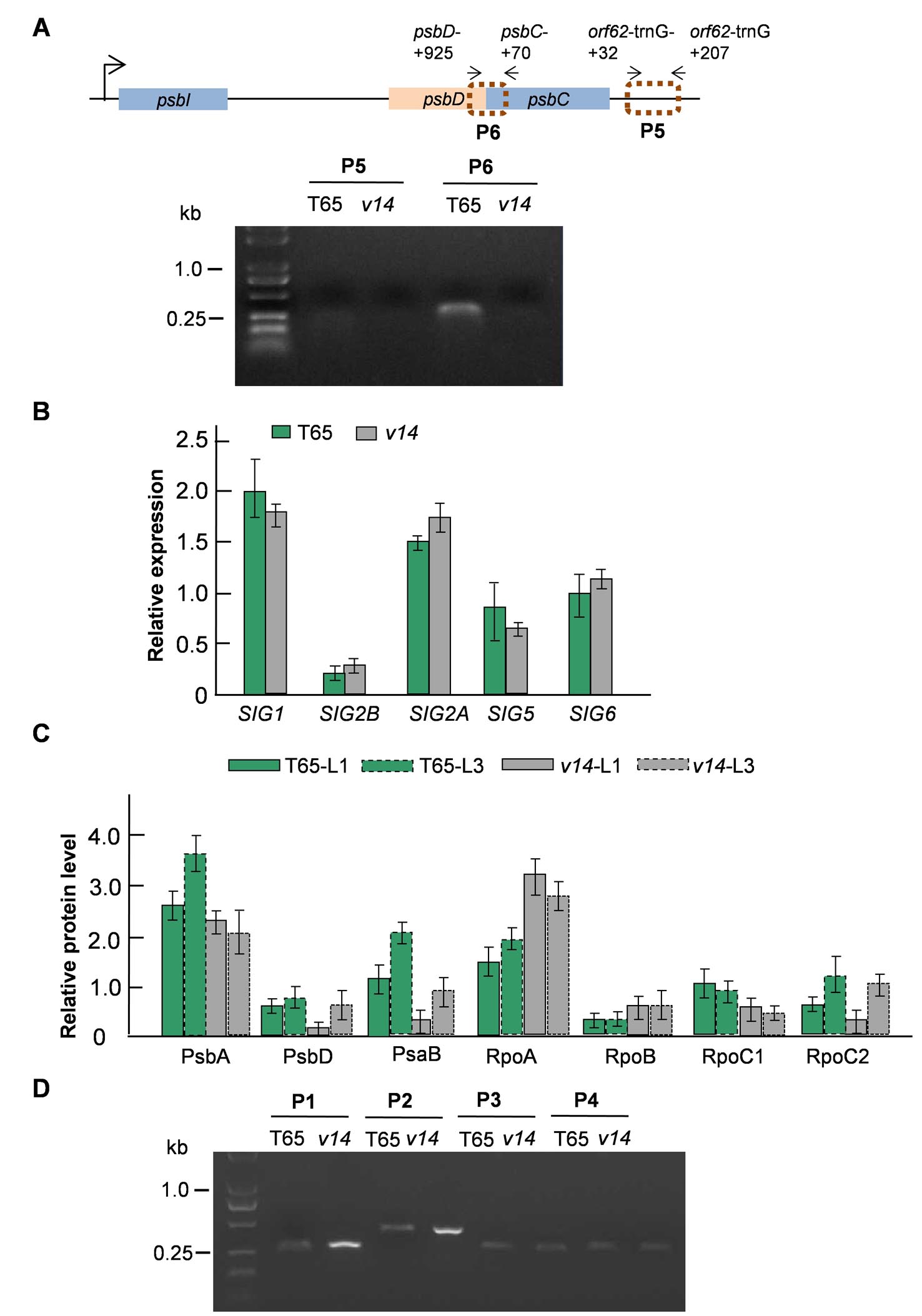

Supplement: Supplementary file 2 — Additional file 2. Analysis of the psbD-containing transcripts by semi-quantitative RT-PCR (31 cycles) (A), expression analysis of the rice sigma factors by qRT-PCR (B), quantification of the immunoblotting analysis shown in Fig. 2B by ImageJ (C), and analysis of the two intercistronic regions (P1 and P2) of rpoB-rpoC1-rpoC2 in the second true leaves (grown at 25 °C) of v14 by semi-quantitative RT-PCR (31 cycles) (D). (A), The positions of the primers are designated relative to the start codon of the ORFs where they are located. (B), The significance compared to T65 was analyzed by t test (n = 3). (C), All the protein levels were normalized to NdhF. (D), The two intercistronic regions (P3 and P4) of psaA-psaB-rps14 were used as the control. (A) and (D), The images presented here are the representatives of three biological repeats. [file 12870_2021_3192_MOESM2_ESM.jpg]

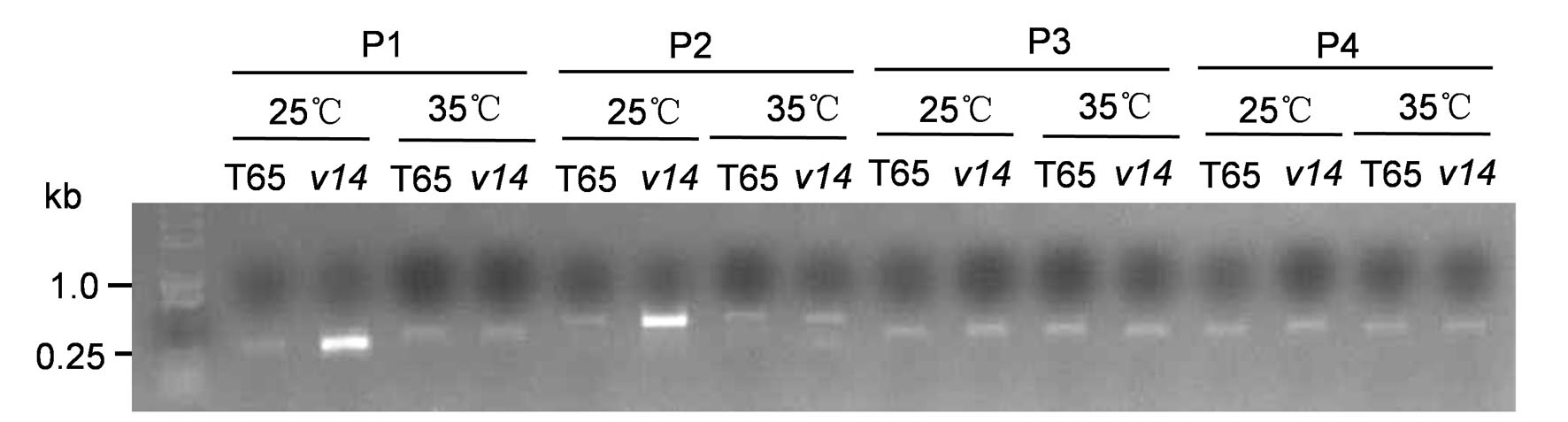

Supplement: Supplementary file 3 — Additional file 3. High temperature (35 °C) rescued the cleavage of the two intercistronic regions (P1 and P2) of the rpoB-rpoC1-rpoC2 precursor in L1 of v14. The semi-quantitative RT-PCR was carried out by 31 cycles. L1, the first leaf. P3 and P4 are the two spacer regions in the psaA-psaB-rps14 operon shown in Fig. 2C. The image presented here is the representative of three biological repeats. [file 12870_2021_3192_MOESM3_ESM.jpg]

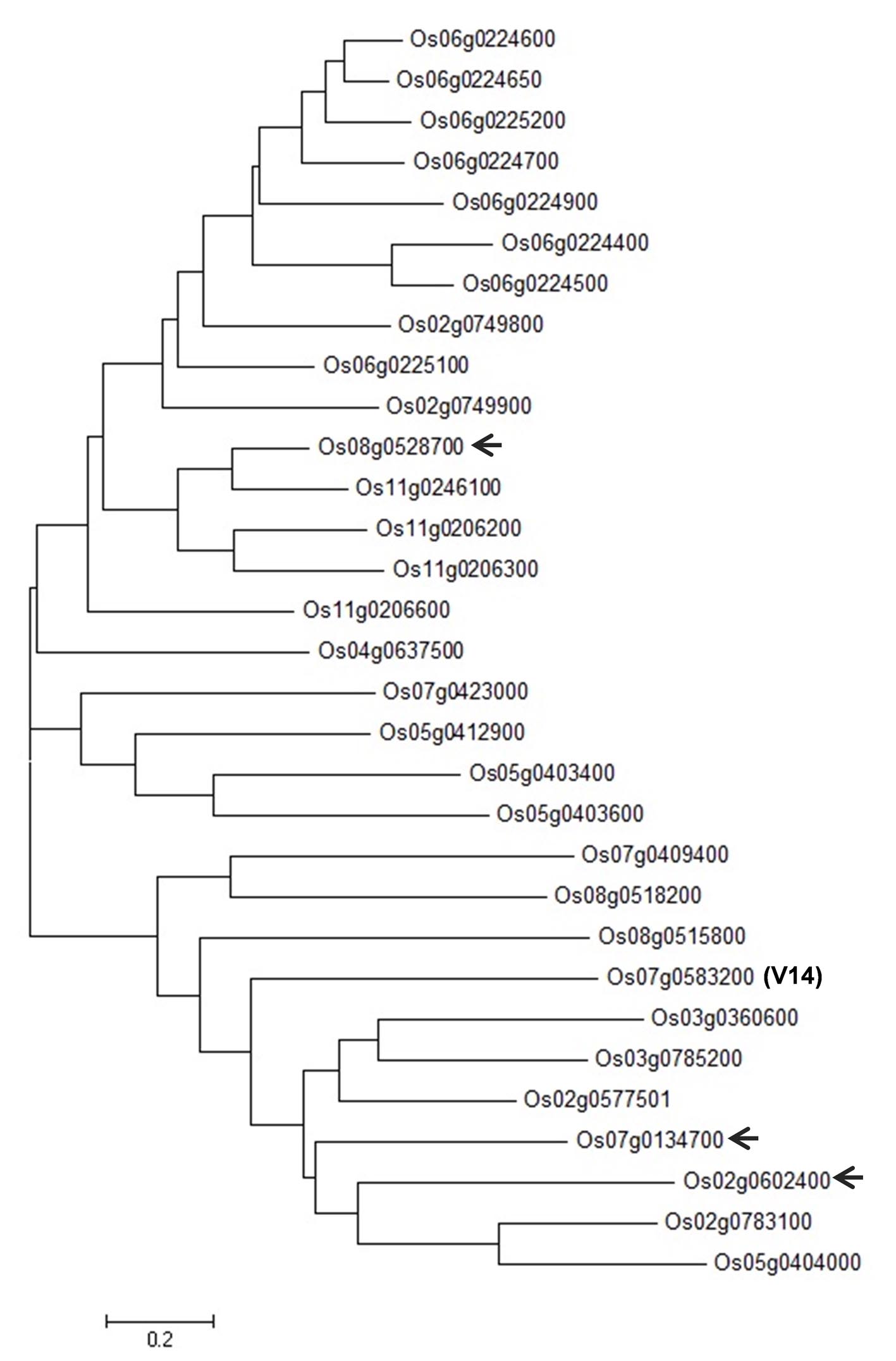

Supplement: Supplementary file 4 — Additional file 4. Phylogenetic analysis of V14 in rice. The neighbor-joining tree was built on protein sequences using the software PHYLIP (version 3.66) and visualized with the software TreeView and MEGA5. Arrows indicate the three genes with temperature-sensitive expression in the second leaf (Fig. 4). [file 12870_2021_3192_MOESM4_ESM.jpg]

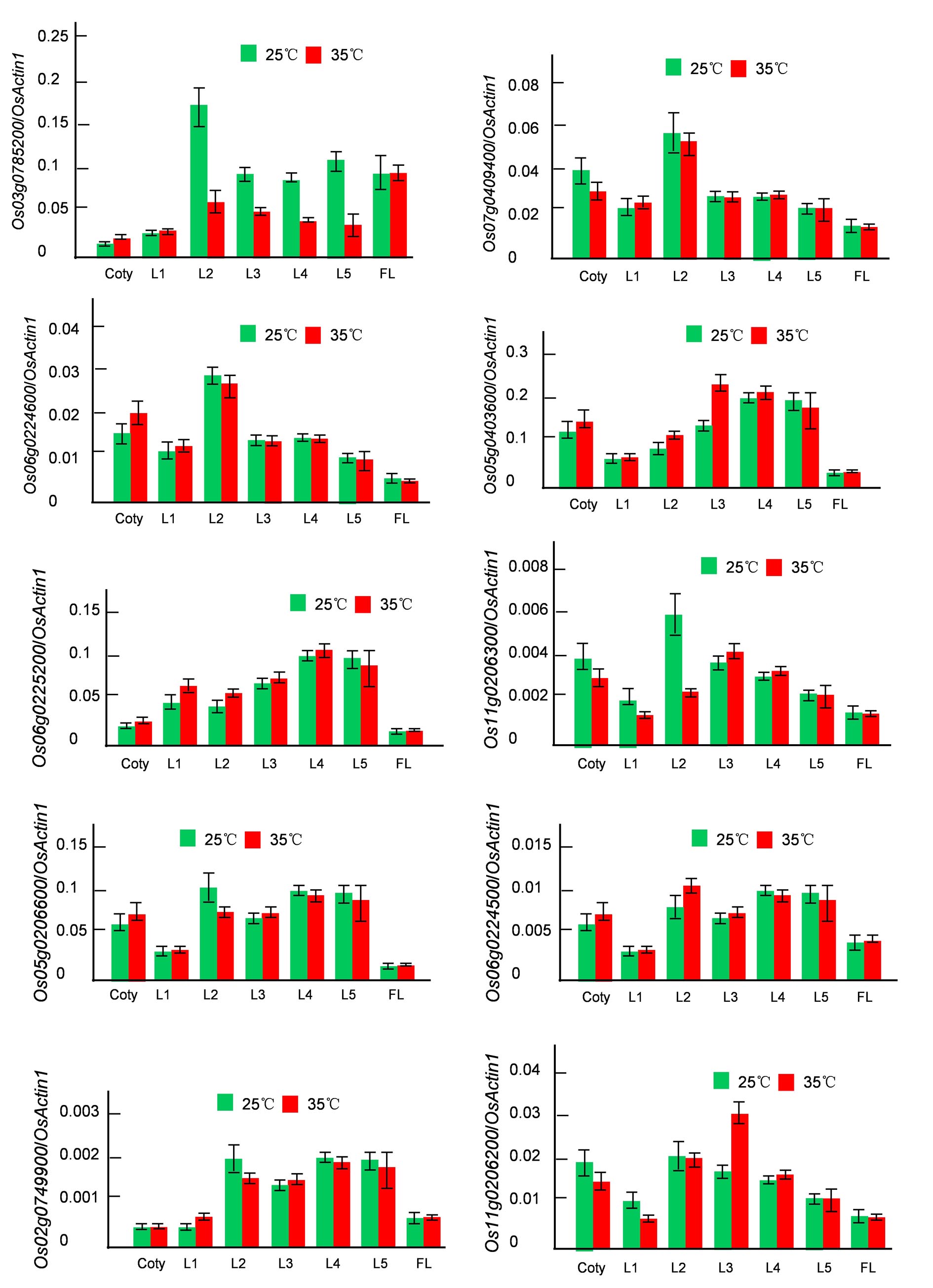

Supplement: Supplementary file 5 — Additional file 5. Gene expression profiles of the other 27 V14-homologous genes at different stages of leaf development at 25 °C and 35 °C. The relative expression levels shown here are the averages of three independent experiments. [file 12870_2021_3192_MOESM5_ESM.jpg]

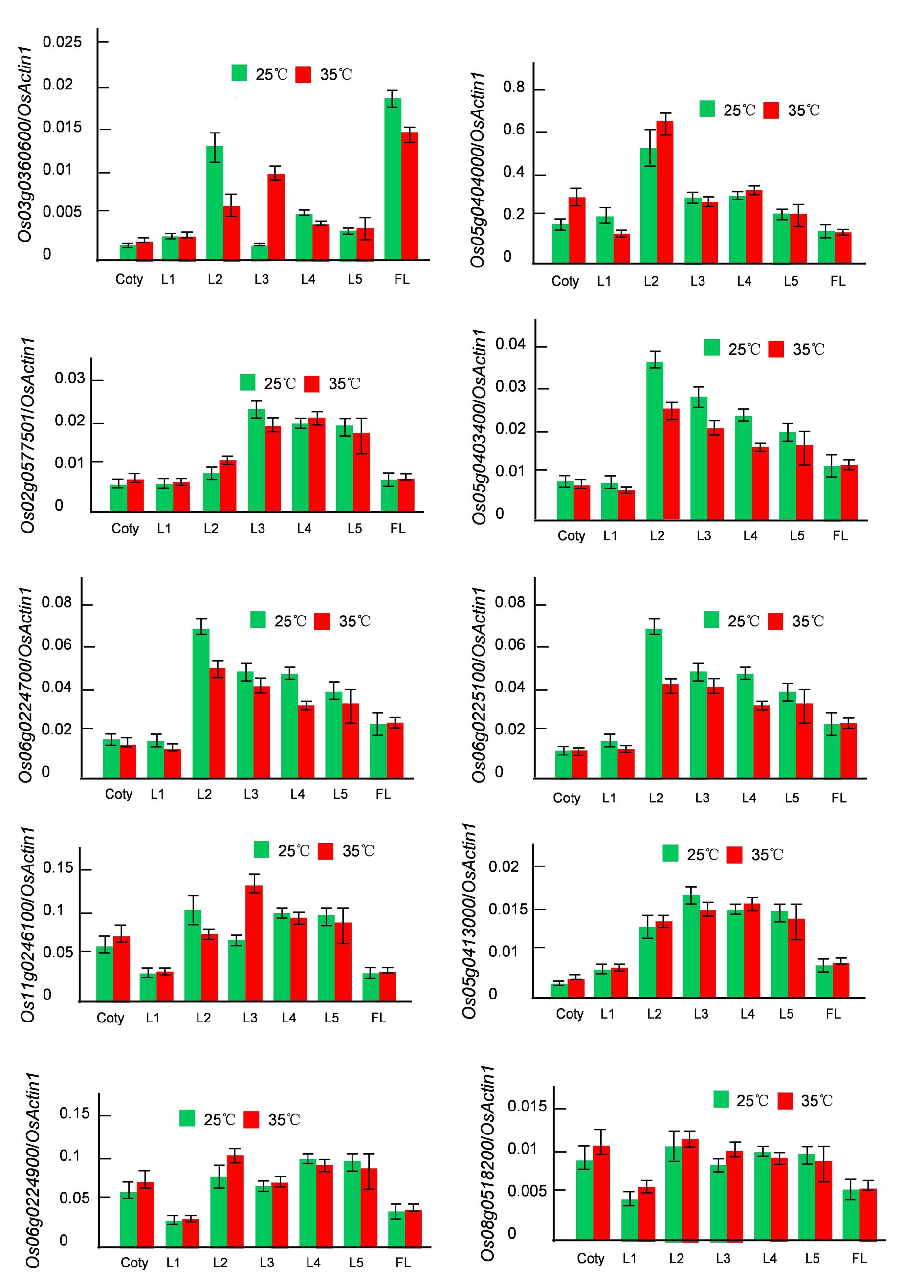

Supplement: Supplementary file 6 — Additional file 6. Gene expression profiles of the other 27 V14-homologous genes at different stages of leaf development at 25 °C and 35 °C. The relative expression levels shown here are the averages of three independent experiments. [file 12870_2021_3192_MOESM6_ESM.jpg]

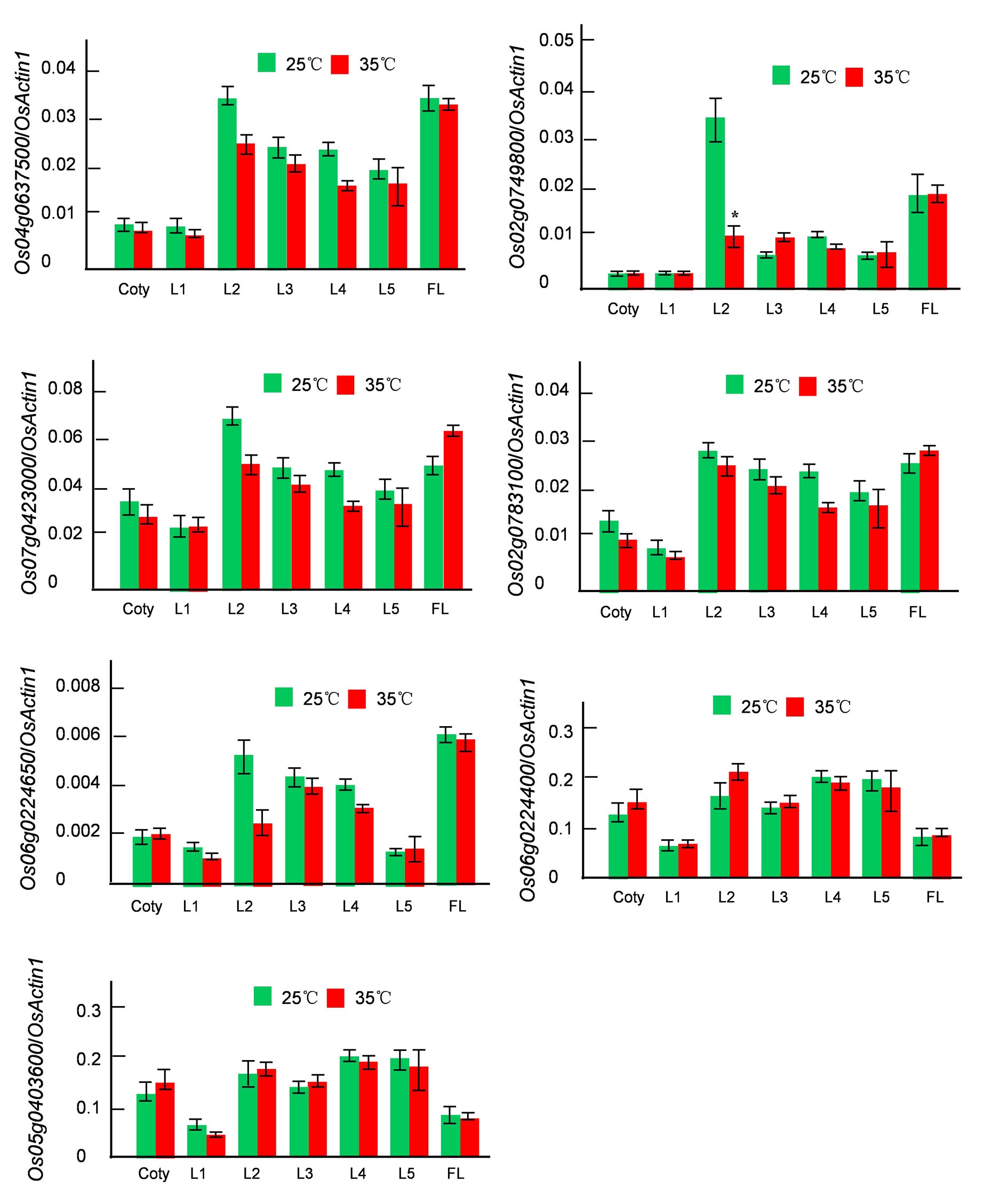

Supplement: Supplementary file 7 — Additional file 7. Gene expression profiles of the other 27 V14-homologous genes at different stages of leaf development at 25 °C and 35 °C. The relative expression levels shown here are the averages of three independent experiments. [file 12870_2021_3192_MOESM7_ESM.jpg]

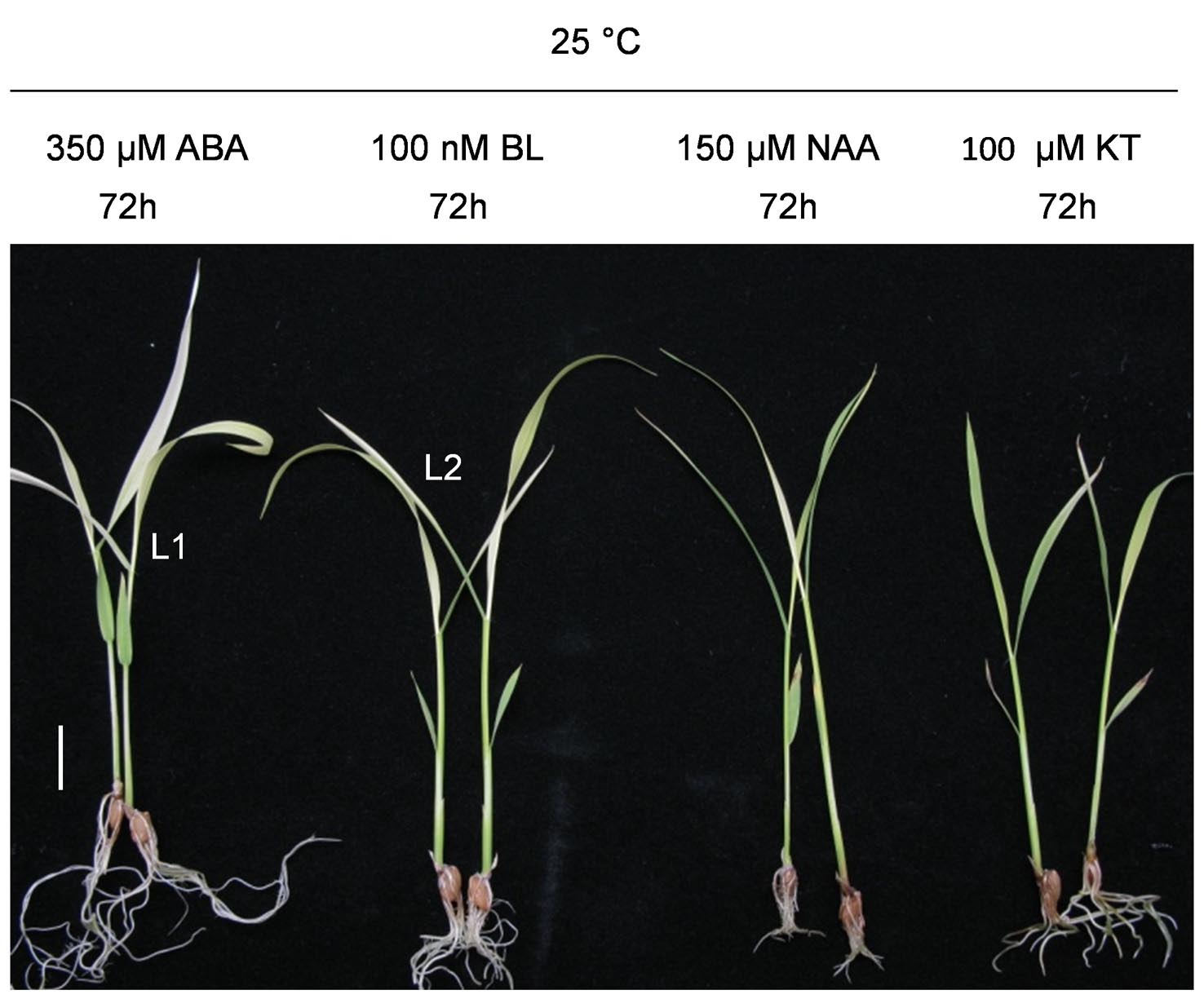

Supplement: Supplementary file 8 — Additional file 8 v14 seedlings on various phytophormone treatments at 25 °C. Bar = 0.5 cm; L1, the first true leaf; L2, the second true leaf. [file 12870_2021_3192_MOESM8_ESM.jpg]

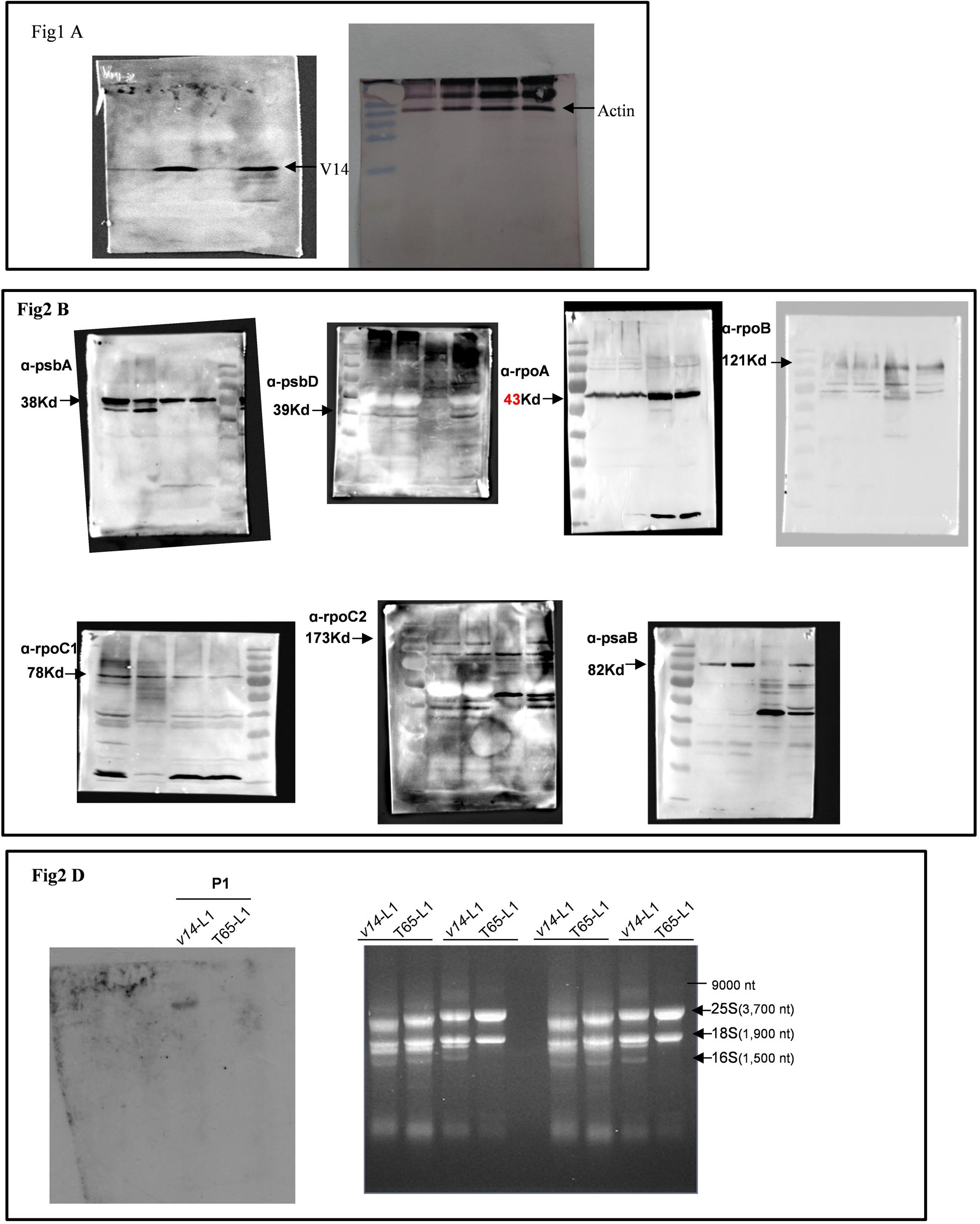

Supplement: Supplementary file 11 — Additional file 11. The un-cropped blot images of Figs. 1A, 2B, and D. [file 12870_2021_3192_MOESM11_ESM.jpg]
